# Inferior vena cava thrombus secondary to blunt abdominal trauma

**DOI:** 10.1259/bjrcr.20160117

**Published:** 2017-04-21

**Authors:** Chadi Diab, Anthony Abou Karam, Shaked Laks, Noemi Brunner

**Affiliations:** ^1^Department of Radiology, Texas Tech University Health Sciences Centre, el paso, TX, USA; ^2^Department of Radiology Imaging Fellowship Program Director Imaging, Bellevue Hospital Center, Newyork, NY, USA

## Abstract

A 15-year-old female presented to the emergency department of a level 1 trauma centreafter being involved in a high-speed motor vehicle accident. The patient underwent a contrast-enhanced CT scan of the abdomen and pelvis obtained with a 60–70 s delay as part of the institution’s polytrauma protocol. The CT scan demonstrated multiple hepatic lacerations and a filling defect in the suprahepatic inferior vena cava adjacent to the cavoatrial junction. Inferior vena cava thrombus secondary to blunt abdominal trauma is extremely rare, and to our knowledge, this is the first reported case of acute thrombus diagnosed by CT at the time of initial injury. There is limited literature on management of this entity. Possible treatments range from conservative approaches to anticoagulation and placement of IVC filters.

## Case report

A 15-year-old female presented to the emergency department of a level 1 trauma centre after being involved in a high-speed motor vehicle accident. After the initial survey was performed in the emergency department, the patient was found to be haemodynamically stable. She underwent a routine whole body scan as part of our institution’s polytrauma protocol. This includes a CT scan of the head and cervical spine followed by contrast-enhanced CT scan of the chest, abdomen and pelvis with reconstructions of the thoracic and lumbar spine. The contrast-enhanced examinations are obtained at the standard 60–70 s delay.

**Figure 1.**
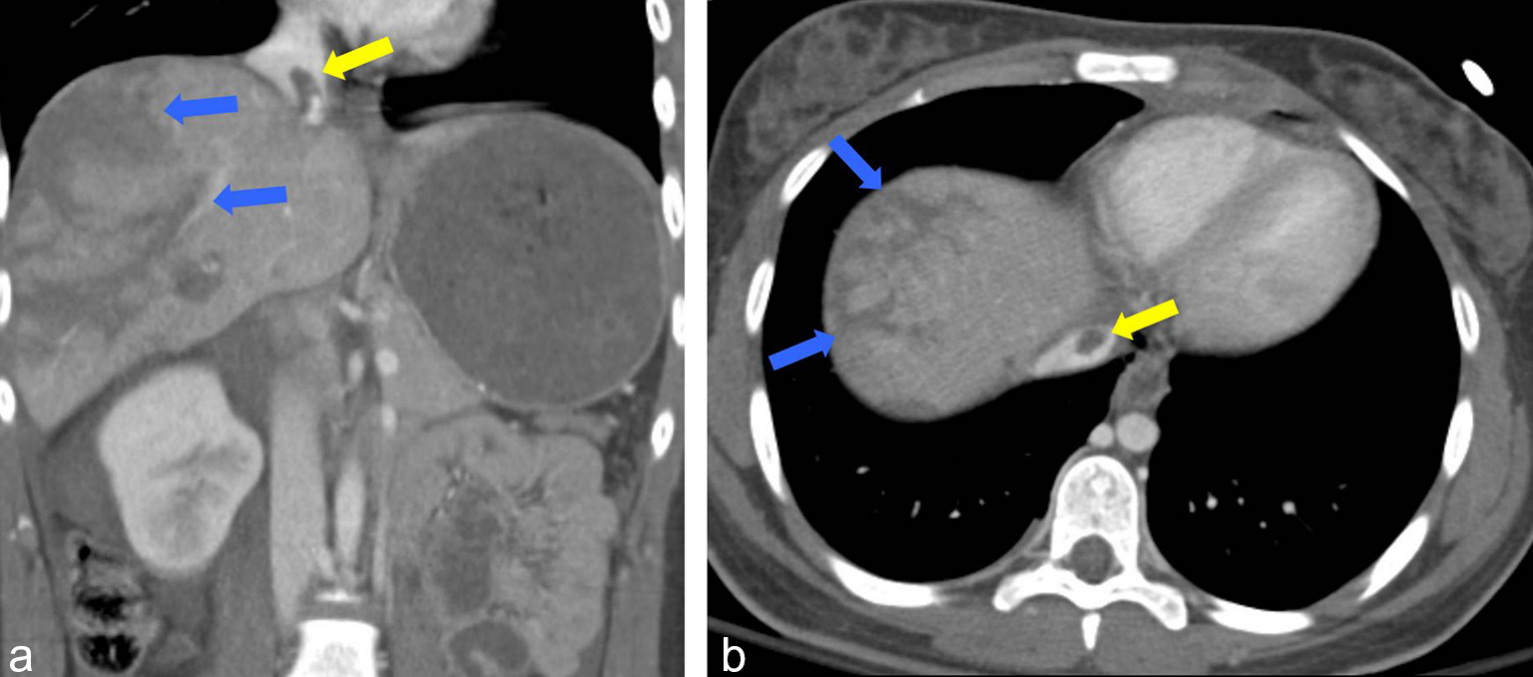
Contrast-enhanced CT abdomen coronal (a) and axial (b) reformats demonstrate the inferior vena cava thrombus (yellow arrow) and the hepatic lacerations (blue arrows).

**Figure 2.**
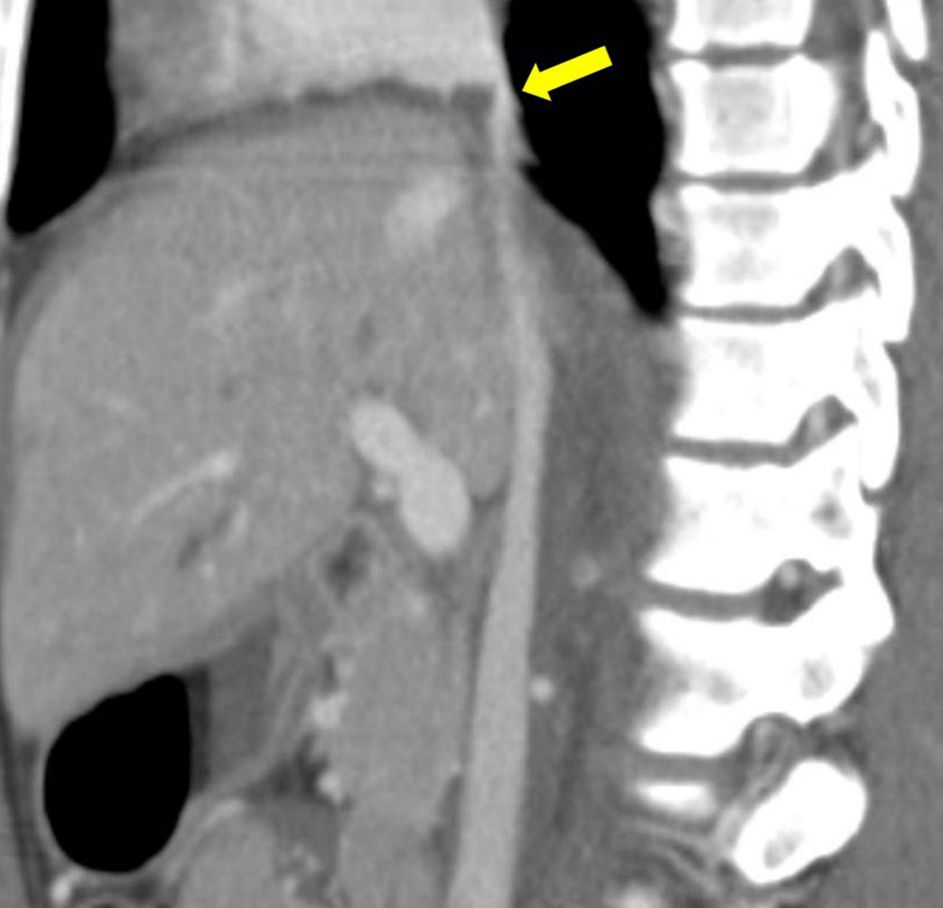
Contrast-enhanced CT abdomen sagittal reformat demonstrates the inferior vena cava thrombus (yellow arrow).

The CT of the abdomen and pelvis demonstrated Grade 5 (AAST liver injury scoring scale) liver lacerations in the right lobe extending to the right and middle hepatic veins (Figure 1a), a centrally located hypodense filling defect in the suprahepatic inferior vena cava (IVC) (Figures 1a,b) extending to the base of the right atrium (Figure 2), a right adrenal gland haematoma, a moderate haemoperitoneum and a grade 4 right kidney injury. Additional findings noted on CT scan of the chest included multiple rib fractures, lung contusions and bilateral small pneumothorax.

During the first days of admission, initial goals were to monitor and manage the early complications of severe polytrauma including respiratory or haemodynamic decompensation, bleeding and coagulopathy. Following admission, the patient required multiple transfusions including packed red blood cells, fresh frozen plasma and platelets. The haemoglobin trended down from 12.3 g dl^−1^ on day 1 to 6.5 g dl^−1^ on day 3 (normal: 12–15 g dl^−1^). She developed consumption thrombocytopenia and the platelet count decreased from 222 000 ul^−1^ on day 1 to 87 000 ul^−1^ on day 3 (normal: 150 000–450 000 ul^−1^). She maintained a mildly elevated INR 1.2–1.7 (normal: 0.8–1.1), a normal partial thromboplastin time 24–30.9 (normal: 23.3–38.6) and mildly elevated fibrinogen levels of 432–720 (normal: 234–500). The severity of the concomitant injuries and the patient’s haemodynamic status precluded considering therapeutic anticoagulation or more invasive interventional or surgical thrombectomy procedures at this time.

A repeat contrast-enhanced CT of the abdomen and pelvis on day 3 of admission demonstrated increased haemoperitoneum, stable solid organ injuries and IVC thrombus without active bleeding. The patient’s haemodynamic status and haemoglobin levels stabilized by day 5 and prophylactic low molecular weight heparin were started. An abdominal ultrasound was performed on day 6 to evaluate the IVC and did not demonstrate an intraluminal thrombus. The patient was noted to have intermittent macroscopic haematuria, and in the absence of clinical or laboratory signs of IVC thrombus progression (back pain, chest pain, oxygen desaturation on room air, lower extremity oedema, rising liver function tests) a decision was made to hold on starting therapeutic anticoagulation. The patient remained stable and was discharged home on day 10. She had no signs or symptoms to suggest progression of thrombosis on a scheduled 4-week outpatient follow-up.

## Discussion

Most typical polytrauma protocols include a portal venous acquisition of the abdomen and pelvis with a 60–85 s delay, which offers good sensitivity for detection of most parenchymal injuries. Additional delayed images (5–10 min) may be obtained in cases where portal venous or urinary tract injuries are suspected.^1^ Although not as widely accepted, the protocol used for trauma patients in some institutions includes an arterial phase and delayed venous phase acquired at 90 s.^2^ Our institution’s standard polytrauma protocol includes portal venous phase images of the abdomen and pelvis, acquired at a fixed 60–70 s delay following i.v. administration of non-ionic iodinated contrast at a rate 3.5 cc s^−1^. Adult patients routinely receive 100–150 cc of contrast while paediatric patients receive a weight-based dose of 2 cc kg^−1^.^3^

Although rare, traumatic thrombosis of suprahepatic IVC should be detected during most routine trauma CT protocols. The suprarenal IVC is best opacified at 60–70 s acquisition after contrast administration owing to venous return from the kidneys.^1^ This coincides well with the standard acquisition of most trauma CTs of the abdomen and pelvis, which are obtained in the portal venous phase. In our case, as expected, the intraluminal filling defect in the suprahepatic IVC was easily detected. However, in cases of suspected IVC thrombosis at the level of the renal vein it is recommended to obtain additional delayed images to differentiate thrombus from contrast mixture.^1^

To our knowledge, this is the first reported case of acute IVC thrombus formation where diagnosis was made by CT at the time of initial injury. Although 12 other cases of IVC thrombus caused by blunt trauma have been previously reported, all occurred or were detected at least 14 days after the initial injury.^4^ Several different mechanisms of IVC thrombosis after blunt trauma have been proposed and may explain a delayed presentation: Injury of the endothelium resulting in secondary thrombus formation, retroperitoneal haematoma compressing the IVC and hepatic vein thrombosis with secondary extension into the IVC. In addition, after trauma, there is a normal physiological response favouring hypercoagulability with suppression of fibrinolysis.^5,6^ The earliest case to be detected was 14 days after trauma in a patient who had a retroperitoneal haematoma, most likely causing extrinsic compression of the IVC.^5^ Some cases were secondary to extension of thrombus from lower extremity deep venous thrombosis.^4^ In most of the cases, the thrombus was associated with liver injuries such as lacerations and haematomas. It is likely that several of the above factors contributed to the rapid IVC thrombosis in our patient who had multiple liver lacerations extending to the right and middle hepatic veins, as well as other traumatic injuries.

While IVC thrombus after blunt trauma may be rare, it has potentially serious complications. The thrombus can be partially or completely occlusive. If located adjacent to the hepatic veins, it can obstruct the venous outflow from the hepatic veins to the IVC. This can induce passive hepatic congestion or acute Budd–Chiari syndrome. The latter might result in acute hepatic failure and death. IVC thrombus can also result in massive pulmonary embolism.^5^ Given the risk of pulmonary embolism, the threshold for obtaining a CT angiogram of the chest should be low if patients develop dyspnoea or hypoxia.

Variable options have been described for the management of these patients including conservative anticoagulation versus more invasive interventional or surgical procedures. While trauma patients are often placed on deep venous thrombosis prophylaxis,^7,8^ there is limited data on therapeutic anticoagulation in patients with liver lacerations. IVC filter is considered a preventive option for thromboembolism in trauma patients with contraindication to anticoagulation due to actively bleeding solid organ injuries.

In cases of known post-traumatic IVC thrombosis, there are two reports of IVC filter placement via the right internal jugular approach, including a suprarenal filter that was retrieved after 8 weeks.^9,10^ Suprarenal IVC filters have also been placed without significant complications in patients with recurrent pulmonary embolism due to non-traumatic IVC thrombosis extending to or above the renal veins and in patients with renal or gonadal vein thrombosis.^11^ Recent advances in filter designs and increasing experience in filter retrieval make suprarenal IVC filters a feasible choice in the correct clinical settings. In our case the suprahepatic location of the IVC thrombosis with extension to the cavoatrial junction precluded the placement of a filter.

In addition, surgical thrombectomy with and without anticoagulation may be considered in cases of massive symptomatic thrombosis or progressive thrombus with contraindication to IVC filter placement or anticoagulation. The size and location of the thrombus play an important role when thrombectomy is being considered. For example, it has been advocated that massive suprahepatic IVC thrombosis warrants emergent thrombectomy.^6^ Thrombectomy was also performed in cases of blunt trauma with IVC thrombosis complicated by acute Budd–Chiari hepatic failure,^12^ extensive ascites with lower extremity oedema and fever^13^ and right upper quadrant pain with unexplained fever and free-floating IVC thrombus.^4^ The interventional hybrid approach of preprocedural IVC filter placement followed by percutaneous mechanical thrombectomy is another potential option in the setting of massive progression of IVC thrombosis, but has not been reported in the settings of trauma.

## Conclusions

Although IVC thrombosis after blunt abdominal trauma is extremely rare, it may result in multiple complications including acute liver failure, pulmonary embolism, ascites, lower extremity oedema and unexplained fever. Cases of post-traumatic IVC thrombus are usually associated with multiple hepatic lacerations and should be readily detected on routine trauma protocol CT abdomen and pelvis obtained in the portal venous phase. Treatment must be tailored on a case by case basis owing to the complexity of the associated injuries and the patient’s haemodynamic status. A simplified approach includes considering anticoagulation when not contraindicated with or without prophylactic placement of an IVC filter. Thrombectomy should also be considered in cases of massive thrombosis, thrombus progression or clinical decompensation.

## Learning points

1. The IVC should be assessed carefully by the radiologist in the setting of blunt trauma, especially in the context of severe hepatic injury or retroperitoneal haematoma.
2. Although trauma protocol CT scans differ by institution, most polytrauma protocols include a portal-venous phase of the abdomen and pelvis, which will readily detect suprarenal IVC thrombosis.
3. Thrombus location and size may affect treatment options. The radiologist should evaluate the burden of IVC thrombus, location in relation to hepatic and renal veins and its effect on the liver including findings of Budd–Chiari or passive hepatic congestion.
4. Retrievable supra or infrarenal IVC filter followed by therapeutic anticoagulation may be a reasonable conservative approach when the patient’s haemostasis allows. Contraindication may include suprahepatic IVC thrombosis and the need for decompression via thrombectomy.

## Consent 

Written informed consent for the case to be published (including images, case history and data) was obtained from the patient(s) for publication of this case report, including accompanying images.
